# Exploring the Mediating Role of Decisional Conflict in the Relationship Between Shared Decision Making, the Use of Routine Outcome Monitoring, Treatment Outcomes and Patient Satisfaction: An Observational Longitudinal Study in Dutch Mental Health Care

**DOI:** 10.1007/s10597-025-01535-z

**Published:** 2025-10-24

**Authors:** Floor Stuit, Fabiana Engelsbel, Annette Boenink, Lotte Barneveld-Oudt, Aartjan Beekman, Margot Metz

**Affiliations:** 1 SynQuest, Cooperation of Mental Health Care Organizations, Leiden, The Netherlands; 2 Amsterdam UMC, Amsterdam, The Netherlands; 3 GGZ Noord-Holland-Noord, Mental Health Care Organization, Heerhugowaard, The Netherlands; 4 Dimence Group, Mental Health Care Organization, Deventer, The Netherlands; 5 GGz Breburg, Mental Health Care Organization, Tilburg, The Netherlands; 6 Tranzo Scientific Center for Care and Wellbeing, Tilburg University, Postbus 90153, 5000 Tilburg, LE The Netherlands

**Keywords:** Shared decision making, Routine outcome monitoring, Therapeutic alliance, Decisional conflict, Mental health care

## Abstract

In Shared Decision Making (SDM) patients and clinicians make joint decisions about treatment. The use of Routine Outcome Monitoring (ROM) as a personalized source of information can be helpful when making decisions together. Research suggests this approach has beneficial effects on treatment outcomes and satisfaction. The study aims to investigate if the factors associated with the SDM process contribute to treatment outcomes and satisfaction and whether these outcomes are mediated by Decisional Conflict. An observational longitudinal study was performed using data from a heterogeneous group of patients treated by three Dutch mental health care organizations; 58 patients completed the first and second measurement. First, regression analyses were conducted to examine the relationship between the application of SDM and use of ROM feedback at the start of treatment and outcome factors Symptom Change, Symptom Severity (measured by Symptom Questionaire-48, Brief Symptom Inventory, 36-Item Short Form Health) and Patient Satisfaction (rating on a scale from 0 to 10) 3 to 6 months later. Second, we conducted mediation analyses to investigate whether Decisional Conflict serves as a possible mediating factor through which the SDM process and the use of ROM feedback influences the outcome variables. Results showed that the use of ROM feedback had a significant effect on Symptom Change, however was not mediated by Decisional Conflict. SDM process variables (Feeling Informed, Clarity and Support) were significantly associated with Symptom Severity. Only the effect of Feeling Informed on Symptom Severity was mediated by Decisional Conflict. Support also accounted for a significant part of the variance in Patient Satisfaction, but there was no mediation effect of Decisional Conflict. The SDM process and the use of ROM feedback appears to play a more important role in improving outcomes than Decisional Conflict. These findings also seem to indicate the importance of the patient-clinician working alliance, because this working alliance influences the SDM process and conversely a good SDM process improves the working alliance. This aligns with the evidence from earlier research. We recommend to continue this research on the impact of decisional conflict using a larger sample over a longer period of time.

## Introduction

Shared Decision Making (SDM) is a collaborative approach in which patients and clinicians make joint decisions about treatment, which has proven to be beneficial in healthcare (Elwyn et al., 2012; Metz et al., 2023). In this approach patients and clinicians share available information from both perspectives and patients are supported to participate actively in decisions about their treatment (Elwyn et al., 2012, 2017a, b). Previous research has shown that, if implemented adequately, the positive effects of this method result in patients that are more informed, satisfied and involved in treatment. SDM could also lead to less decisional conflict (Metz et al., 2018a, b; Westermann et al., 2013), more treatment adherence and better treatment outcomes (Clever et al., 2006; Elwyn et al., 2017a, b; Joosten et al., 2009; Malm et al., 2003; Westermann et al., 2013).

Despite these promising research results, the legal embedding of SDM in the Dutch Medical Treatment Contracts Act (Wet Op de Geneeskundige Behandelingsovereenkomst, 1995) and the general consensus among clinicians and patients regarding its importance (Ubbink et al., 2021), the application of SDM in (mental) health care practice remains insufficient (Dahlqvist Jönsson et al., 2015; Joosten et al., 2009; Joseph-Williams et al., 2014; Metz et al., 2019). These findings suggest that there is room for improvement in the implementation of SDM. Given the complexities and challenges of effectively implementing SDM, it is important to better understand the mechanisms that can enhance SDM and maximize its impact (Metz et al., 2019).

Previous research suggests that a shared understanding of patients’ health and life goals, as well as a constructive patient-clinician working alliance are important aspects of SDM. Patients also report that a good working alliance positively influences their preferences and involvement in treatment decisions (Eliacin et al., 2015a, b; Metz et al., 2023), while SDM itself can further strengthen this relationship (Eliacin et al., 2015b; Marchi et al., 2024; Priebe & Mccabe, 2008). A qualitative study in Dutch mental health care offers specific suggestions for clinicians’ roles in SDM (Metz et al., 2023). Clinicians should focus on listening, informing, fostering equal cooperation, and aligning treatment with the individual wishes and values of patients. Trust, openness and honesty are essential components that patients value in the decision-making process (Metz et al., 2023). However, patients differ in how they value and prefer to engage in SDM (Drivenes et al., 2020; Fisher et al., 2020; Levinson et al., 2005; Thimm et al., 2020). Therefore, clinicians should explicitly communicate that there are choices to be made, discuss the division of roles between patients, their relative(s) and the clinician, and provide information tailored to the patients’ needs. Additionally, clinicians should actively support patients in exploring their values and preferences to facilitate joint decisions about treatment options (Joseph-Williams et al., 2014; Metz et al., 2019; Ubbink et al., 2021).

Routine Outcome Monitoring (ROM) serves as a useful source of information in the SDM process about treatment. ROM involves the repeated measurement of symptom severity, functioning, and well-being, providing data that can be used to evaluate and, if necessary, adjust the treatment plan. Patients complete questionnaires assessing their symptoms and their impact on daily life before, during and at the end of treatment (Barkham et al., 2023; Carlier et al., 2012a, b). The results are then presented visually, helping patients track and identify topics to discuss with their clinician (Metz et al., 2023). Research has shown that using ROM as a personalized source of outcome information can be a helpful tool in SDM (Metz et al., 2019; van der Feltz-Cornelis et al., 2014). Patients find ROM particularly helpful when they can review and discuss the results with their clinician, as it enhances their involvement in the decision-making process (Metz et al., 2023). Additionally, access to their own ROM data supports more informed and meaningful discussions. ROM has shown to improve mental health status, especially for patients who are not responding well to treatment (Delgadillo et al., 2018; Gondek et al., 2016; Gual-Montolio et al., 2020; Jensen-Doss et al., 2020; Lewis et al., 2019; Shimokawa et al., 2010) and it also facilitates the communication between patients and clinicians (Barkham et al., 2023; Jensen-Doss et al., 2020).

To evaluate the quality of SDM, the concept Decisional Conflict can be used (Garvelink et al., 2019; Metz et al., 2018a, b; Westermann et al., 2013). Decisional Conflict explains the degree to which patients are involved in and are satisfied with key clinical decisions. The Decisional Conflict Scale (DCS) assesses five dimensions of decision making from patients’ perspective. Three dimensions focus on the SDM process: Feeling Informed, Having Clarity about values and Feeling Supported. The other two dimensions describe outcomes of the decision-making process providing insight into satisfaction with the decision namely the subscales Feeling Uncertain about choice options and (perceived) Quality of the Decision. In this study, these latter two dimensions are referred to as Decisional Conflict (Metz et al., 2018a, b).

Given the positive potential effects of SDM, the need for improvement in its practical implementation, and the limited understanding of the underlying mechanisms, this observational longitudinal study aims to investigate the relationship between SDM implementation and the use of ROM with the outcome variables Symptom Change, Symptom Severity and Patient Satisfaction. Specifically, we seek to understand the role of satisfaction with the decision -conceptualized as Decisional Conflict- as a possible mediating factor between the SDM process and the outcome measures mentioned above. In particular, this is relevant, because mental health care patients report a higher degree of decisional conflict compared to those in general healthcare (Garvelink et al., 2019; Metz et al., 2018a, b). We hypothesize that a better application of SDM and more use of ROM feedback will lead to better treatment outcomes i.e. Symptom Change as a primary outcome and Symptom Severity and greater Patient Satisfaction during treatment as secondary outcomes. Additionally, we expect that SDM will reduce Decisional Conflict, which in turn will lead to more Symptom Change, less Symptom Severity and higher Patient Satisfaction. Gaining insight into these relationships could enhance our understanding of the mechanisms underlying effective SDM and use of ROM feedback in clinical practice which contributes to their improved implementation.

## Methods

### Participants and Procedure

During the intake assessment or at the start of treatment, new patients receiving ambulatory care at three Dutch mental health care organizations were invited by their main clinician or research assistant to participate in this study. Inclusion criteria were: patients aged 18 or older, proficient in the Dutch language and not experiencing a crisis. In two organizations, patients first received an oral explanation, followed by an information letter and an informed consent. In the third organization, patients first received an email. All participating patients signed an informed consent form.

Participants completed symptom-specific (ROM) questionnaires at both T1 and T2, which measured the symptoms relevant for the patient group. At T1 (between December 2020 and February 2022) patients also completed a questionnaire assessing the extent to which SDM and ROM were implemented during the intake and start of treatment (T1). Participants received a link via email to complete the standard ROM and research questionnaires, with reminders sent by e-mail or phone if necessary. To ensure a diverse representation of patients from different treatment settings within specialist outpatient mental health care, the study sample included a heterogeneous group of patients varying in gender, age, education level, treatment setting and diagnoses.

The Medical Ethics Committee declared that the Medical Research Involving Human Subjects Act (WMO) did not apply to this study (reference number: 2020.474), therefore an official approval was not required. Each organization obtained approval from their own local Scientific Review Boards. The study was reported in accordance with the STROBE Statement (STROBE Initiative, n.d).

### Sample Size

The sample size was determined based on an anticipated small expected effect size for the primary outcome Symptom Change and a medium mediation effect of Decisional Conflict and diagnosis as potential interaction variable. To detect a mediation effect, a minimum amount of 118 patients with completed questionnaires at T1 and T2 was required (Fritz & MacKinnon, 2007).

### Measurements

#### Primary Outcome: Symptom Change

The primary outcome Symptom Change was measured with the standard Routine Outcome Monitoring questionnaire of each participating organization at T1 and T2. From each organization outcomes from one of the following measures were available: Symptom Questionnaire-48 (SQ-48), Brief Symptom Inventory (BSI) or 36-Item Short Form Health (SF-36). Symptom Change (symptom score at T2 minus symptom score at T1), was calculated using Cohen’s effect size (Cohen, 1988). The SQ-48 is a questionnaire consisting of 37 items that measure symptoms in the past week. The remaining questions that focus on work and resilience, were not utilized in this study. Responses were given on a five-point Likert scale, where 0 corresponds to “Never,” 1 to “Rarely,” 2 to “Sometimes,” 3 to “Frequently,” and 4 to “Very often”. Higher scores indicate a greater level of psychological distress. The symptom-related questions were summed in a total psychopathology score ranging from 0 to 148 (Carlier et al., 2012a, b).

The SQ-48 demonstrated good internal consistency, with Cronbach’s alpha ranging from 0.78 to 0.98 across different scales, and good convergent validity (*r* >.56) in studies involving patients with depressive, anxiety or somatoform disorders (Carlier et al., 2012a, b). The total psychopathology score revealed a good temporal stability measured through test–retest reliability (ICC =.93) and satisfactory sensitivity of change (Carlier et al., 2017).

The BSI is a multidimensional questionnaire designed to measure the severity of psychological symptoms experienced by participants over a recent period. The BSI consists of 53 questions which were answered on a five-point Likert scale, ranging from 0 “None at all” to 4 “A lot”. It includes nine subscales that asses specific symptom dimensions: somatization, obsession-compulsion, interpersonal sensitivity, depression, anxiety, hostility, phobic anxiety, paranoid ideation and psychoticism. In this study, the total score of 53 items was used, with higher scores indicating greater psychological distress. The total score demonstrated high reliability (α =.96), and the validity was supported by a strong ability to differentiate between patients and healthy respondents as well as between patient groups with different diagnoses (de Beurs & Zitman, 2006).

The SF-36 is a multipurpose participant-reported survey aimed at measuring health related quality of life. It contains 36 questions across 8 subscales that evaluate the following domains: physical functioning, role limitations due to physical health, limitations due to emotional problems, energy/fatigue, emotional well-being, social functioning, pain, general health and health change. This questionnaire was selected for patients with somatoform disorders because it captures both physical and mental health aspects. Respondents gave answers to questions covering a four-week period. To compute a total score, a substantial part of the raw scores (items 3 to 19, 24, 25, 28, 29, 31 to 33, 35) were recoded, summed, and transformed in a scale ranging from 0 to 100, with higher score indicating better health status (RAND Corporation, 2023). Considerable evidence supported the reliability of the SF-36 as well as its validity, shown by the effectiveness of the SF-36 in distinguishing between groups with known health differences (Brazier et al., 1992). Among Dutch patients and non-patients, the reliability was found to be very high (α =.84). Cronbach’s alpha, measured among Belgian and French patients, was found to be good (α >.80) (Razaval & Gandek, 1998; Viane, 2002; Ware & Sherbourne, 1992).

#### Secondary Outcomes

In addition to the primary outcome Symptom Change, two secondary outcomes were used: Symptom Severity and Patient Satisfaction at T2, to allow for further exploration. Symptom Severity was measured as sum scores at T2, converted to Z-values to indicate Symptom Severity relative to the study population (within the SQ48, BSI or SF-36 group), and patient satisfaction as a self-reported rating. Regardless of Symptom Severity at the start of treatment, the level of SDM and the use of ROM might have influenced Symptom Severity and Patient Satisfaction during treatment at T2. Depending on the organization, Symptom Severity was measured using the SQ-48, BSI or SF-36. Patient Satisfaction was measured using a scale from 1 to 10, with the question: “How would you rate your treatment on a scale from 1 to 10?”, where 1 indicates very poor and 10 indicates excellent.

#### Application of ROM and Decision Being Made

To assess the extent to which ROM was applied as a personalized source of information in the SDM process, patients answered five questions about the use of ROM feedback (Metz et al., 2015), for example: “My clinician told me that he wants to use the ROM results to make appropriate choices with me about what will happen in the treatment”. The first four items were rated on a scale from 0 (strongly disagree) to 5 (strongly agree), with higher scores indicating better use of ROM feedback. The fifth question is a ‘yes’ or ‘no’ question, asking whether decisions were about diagnosis, type of treatment, setting (individual group, outpatient, part-time, clinic), treatment plan with goals, number of sessions and duration of treatment, daily activities (living/working/learning), continuation or completion of treatment, relapse prevention or other decisions. If a decision was made, patients were directed to the next questionnaire; if not, no questions about SDM and Decisional Conflict followed.

#### Process of Shared Decision Making

**Fig. 1:**
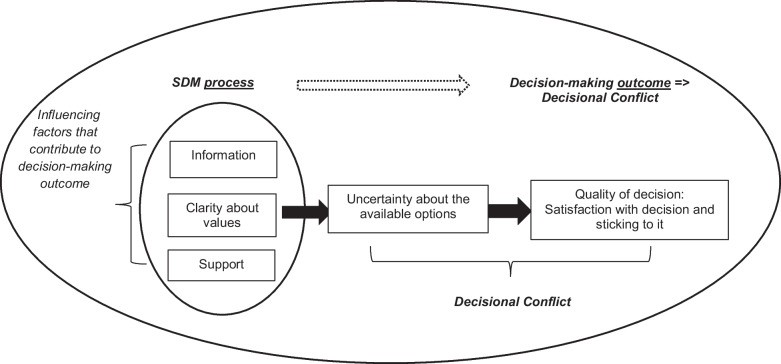
The decisional conflict scale: SDM process and decisional conflict

The process of SDM was evaluated by using three subscales from the Decisional Conflict Scale (Fig. 1) (DCS) (Garvelink et al., 2019; Metz et al., 2018a, b; Westermann et al., 2013) Information, Clarity about Values and Support. Each subscale consists of three questions (Table 1), such as “I know what options are available to me”, which is part of the Information subscale. These nine items were selected to assess the degree to which SDM was applied from the patient’s perspective (Metz et al., 2018a, b). Responses were rated on a scale from 0 (strongly agree) to 5 (strongly disagree), with a higher score indicating less effective SDM. To calculate the scores for the three dimensions, individual item scores were summed, divided by the number of items and multiplied by 25, resulting in a score ranging from 0 to 100. The English version of the DCS, validated in general health care, demonstrated good test–retest reliability (ICC =.81) and adequate Cronbach’s alpha coefficients (α >.78) for the overall score and individual dimensions. The scale’s construct validity, responsiveness to change, and predictive validity of the English DCS was confirmed (O’Connor, 1993). Internal consistency was good for the Information and Clarification of Values subscales (α =.88), but the ‘Support’ subscale shows an insufficient internal consistency (α =.66) (Metz et al., 2018a, b).

**Table 1:** Decisional conflict scale: questions per subscale

Decision-making process
Information
I know which options are available to me
I know the benefits of each option
I know the risks and side effects of each option
Clarity about Values
I am clear about which benefits matter most to me
I am clear about which risks and side effects matter most
I am clear about which is more important to me (the benefits or the risks and side effects)
Support
I have enough support from others to make a choice
I am choosing without pressure from others
I have enough advice to make a choice
Decision-making outcome = > Decisional Conflict
Uncertainty
I am clear about the best choice for me
I feel sure about what to choose
This decision is easy for me to take
Quality of the decision
I feel I have made an informed choice
My decision shows what is important to me
I expect to stick with my decision
I am satisfied with my decision

#### Decisional Conflict

To determine the degree of Decisional Conflict (Fig. 1) from the patient’s perspective, the Uncertainty and Quality of the Decision subscales of the DCS were used (Table 1) (Metz et al., 2018a, b). The scores from these subscales were summed, divided by the number of items, and multiplied by 25, also resulting in a score from 0 to 100. High scores indicate greater levels of Decisional Conflict, which is considered negative. The Uncertainty subscale, which includes three questions, measures the (un)certainty about options to choose from, such as “I am clear about the best choice for me”. The Quality of the Decision subscale consists of four questions measuring the perceived quality or efficacy of the decision making, for example, “I feel I have made an informed choice”. Both subscales demonstrated good internal consistency, with alpha coefficients of α =.84 for Uncertainty and α =.85 for Quality of the Decision (Metz et al., 2018a, b).

#### Demographics

Demographic data (gender, age, education level) and clinical information (treatment setting and diagnosis) data were extracted from the electronic patient files of the three participating organizations.

### Statistical Analysis

#### Descriptives

Socio-demographical and clinical characteristics were calculated using descriptive statistics (N, percentage, mean, SD). Correlations between the independent variables (Information, Clarity of Values, Support and use of ROM) were calculated to determine whether these subscales should be treated as a single independent variable or as four separate independent variables.

**Fig. 2:**
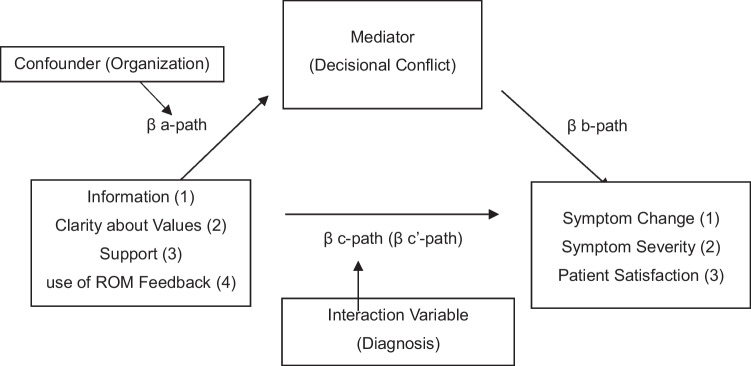
Conceptual model illustrating the impact of SDM elements (Information, Clarity about Values, Support, and use of ROM Feedback) on symptom change, symptom severity, and patient satisfaction mediated by decisional conflict (DC)

Mediation analyses, based on the framework of Baron & Kenny (1986) were conducted to examine whether elements of the SDM process at T1 had a direct effect on the primary outcome Symptom Change and secondary outcomes (Symptom Severity and Patient Satisfaction at T2), and whether this effect was mediated by DC (Fig. 2). Four independent variables representing different elements of the SDM process were examined separately: 1) Information 2) Clarity about Values 3) Support and 4) the use of ROM feedback. The first three variables were analyzed individually because combining the three subscales of the Decisional Conflict Scale would result in a loss of potential discriminating information (Metz et al., 2018a, b). The fourth independent variable, use of ROM feedback, was not correlated to the three subscales mentioned before and therefore also analyzed separately. Therefore, four mediation analyses were conducted for each outcome variable. To correct for the risk of a type I error, a Bonferroni correction was applied, dividing the p-value of.05 by the number of predictors (four), leading to a significance level of *p* =.0125 for each of the four mediation analysis within each outcome.

All mediation analyses involved the examination of four paths (Fig. 2): i) The a-path, determining if the independent variables significantly predicts the mediator; ii) The b-path, investigating if the mediator significantly predicts the dependent variables when controlling for the independent variables; iii) The c-path, assessing if the independent variables significantly predict the dependent variables without considering the mediator; iv) The c’-path, evaluating if the direct effect of the independent variables on the dependent variables remain significant while controlling for the mediator. If paths a, b, and c are (marginally) significant, and the direct effect (path c') is reduced or becomes non-significant when controlling for the mediator, this means that the effect of the independent variable on the dependent variable is mediated by the mediator Decisional Conflict.

The described paths were investigated through two distinct regression analyses. A linear regression was conducted to evaluate the a-path. Following this, a hierarchical multiple regression analysis was performed to simultaneously assess the b-path, c-path, and c’-path. In this multiple regression, the independent variables were included in Block 1, while the mediator and independent variables were added together in Block 2, using the ENTER method. The total indirect path, represented by the product of paths a and b (ab), quantifies the overall mediation effect of the mediator variable on the relationship between the independent variables and the dependent variables. To evaluate if this indirect effect is significant, a Sobel test (MacKinnon et al., 2004) was performed (Soper, n.d.).

This analysis was applied to the primary and secondary outcome variables: 1) Symptom Change between T1 and T2 (T2-T1), 2) Symptom Severity (at T2) and 3) Patient Satisfaction (at T2). Prior to these analyses, linearity between the proposed variables in our model was assessed with deviation from linearity surpassing *p* >.05 and with a visual inspection of scatterplots. Reliability was calculated with Cronbach’s alpha for each of the subscales of the DCS and the questions regarding the use of feedback.

#### Confounding and Interaction Effects

In the regression analyses mentioned above, the potential confounder ‘mental health care organization’ was first included in the a-path, as the implementation of SDM and use of ROM feedback might vary between the participating mental health care organizations (Fig. 2). Second, the potential interaction variable diagnosis type was added to the c-path (diagnosis), since the diverse diagnostic groups could potentially influence the relation between independent and dependent variables (Fig. 2). The data from the participating organizations revealed three diagnostic clusters: mood disorders (depressive and bipolar disorders), personality disorders and a third category labeled as remaining (i.e. somatic unexplained physical complaints, anxiety, eating disorders and post-traumatic stress disorders). These variables were transformed into dummy variables for inclusion in the regression analysis. To correct for a type I error, a Bonferroni correction was applied (dividing the p-value of.05 by the number of predictors, which is four). This resulted in a significance level of *p* =.0125 for each of the four mediation analysis within each outcome. To determine whether a variable was a confounder, the following rule of thumb was used: the organization variable needed to explain more than 10% of the variation in a-path. To assess whether diagnosis was an interaction variable in the c-path, the aforementioned significance level was used. Data were analyzed with Statistical Package for the Social Sciences (SPSS) version 29).

## Results

### Participants

**Fig. 3:**
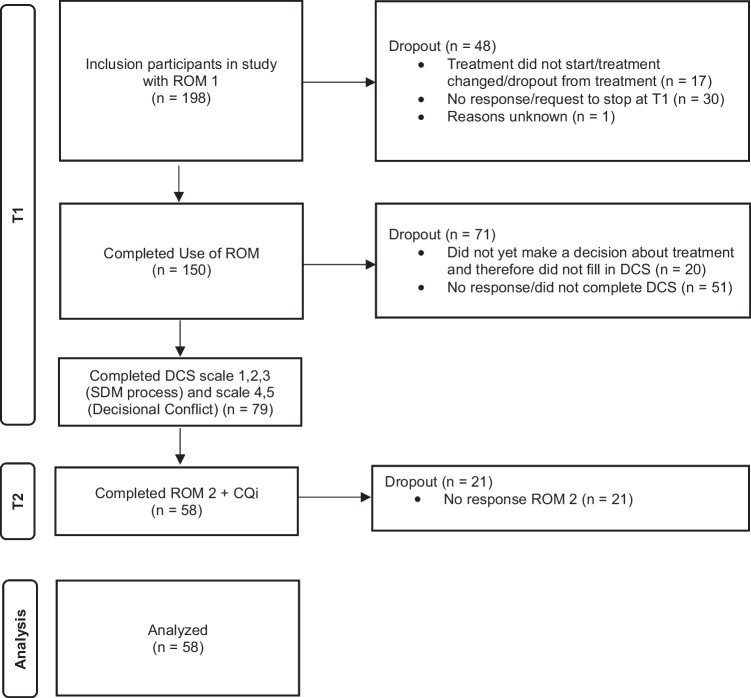
Flowchart of the inclusion process and drop-out

Data were collected from three Dutch specialist mental health care organizations with an initial sample of 198 participants that. However, only participants with complete data at both T1 and T2 were included in the analyses. The flow chart of the study (Fig. 3) illustrated the number of and reasons for dropouts.

### Descriptives

Sample characteristics of the included patients are presented in Table 2. Participants (*N* = 58) had a mean age of 37, ranging from 18 to 60 years, and were mostly female (73%). The most commonly reported diagnoses were depressive disorders, personality disorders and anxiety disorders.

**Table 2:** Participant characteristics

		% (*N*)	M (SD)
Gender	Female	74 (43)	
	Male	26 (15)	
Age		100 (58)	37(12)
Education	Low	12 (7)	
	Intermediate	52 (30)	
	High	30 (18)	
	Missing	6 (3)	
Diagnosis	Depressive disorders	36 (21)	
	Personality disorders	33 (19)	
	Other:		
	Anxiety disorders	10 (6)	
	Trauma and stressor related disorders	7 (4)	
	Obsessive–Compulsive and related disorders	5 (3)	
	Somatic symptom and related disorders	2 (1)	
	Bipolar disorders	2 (1)	
	Missing	5 (3)	
Organization and ROM Questionnaire	1. GGz Breburg (SQ-48)	72 (42)	
	2. Dimence Group (BSI)	24 (14)	
	3. Amsterdam UMC (SF-36)	4 (2)	

*M* mean *SD* standard deviation

Descriptives of the independent, potential mediator and dependent (outcome) variables are shown in Table 3. The primary outcome, Symptom Change, revealed a decrease in complaints over the course of the intervention, with a small to medium effect size of *d* = -.39 between ROM 1 and ROM 2 with an average time interval of 146 days between measurements. Bivariate correlations reported moderate to strong correlations between the subscales of DCS about the SDM-process (Subscales 1 and 2: *r* =.77; subscales 2 and 3: *r* =.58, and subscales 1 and 3: *r* =.62). Application of ROM showed weak correlations with the DCS scales (Subscale 1: *r* = -.18; subscale 2: *r* = -.21, and subscale 3: *r* =.16). Internal consistencies of the SDM subscales ranged from sufficient to good (Information: α =.875; Clarity of Values: α =.846; use of ROM feedback: α =.725), except for Support: (α =.659). The internal consistency of the possible mediator Decisional Conflict is good (α =.926). Regarding the type of decision made, most participants reported decisions about the type of treatment (*N* = 41), followed by decisions about diagnosis (*N* = 10).

**Table 3:** Descriptives decisional conflict scale

	M (SD)
IV 1: DCS subscale 1 Information (*N* = 58)	38.07 (19.81)
IV 2: DCS subscale 2 Clarity about Values (*N* = 58)	39.80 (19.12)
IV 3: DCS subscale 3 Support (*N* = 58)	32.18 (18.76)
IV 4: use of ROM feedback (*N* = 58)	2.21 (1.18)
Mediator: Decisional Conflict (*N* = 58)	33.68 (19)
DV 1: Symptom Change: Effect size scores ROM 2 minus ROM 1 (*N* = 58) Time between ROM 2 and ROM 1 in days	−0.3883 (1.01)
	146.03 (57.26)
DV 2: Symptom Severity: z-values T2 (*N* = 58)	0 (0.98)
DV 3: Patient Satisfaction: Patients grade T2 (*N* = 58)	7.25 (2.06)

*M* mean *SD* standard deviation

### Regression/Mediation Analysis

#### Primary Outcome Symptom Change

**Fig. 4:**
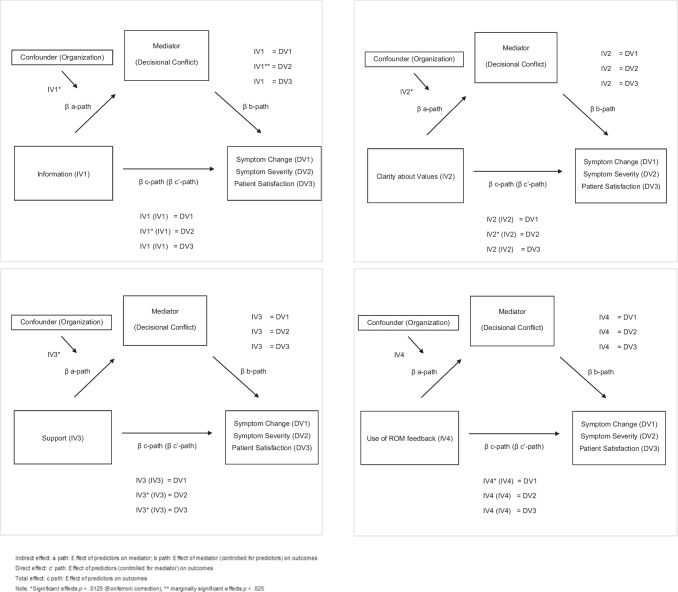
Mediation analysis and significant effects IV1-IV4

We hypothesized that better application of SDM and the use of ROM feedback would lead to a reduction of symptoms at T2, both directly and indirectly through a decrease in Decisional Conflict. A linear regression analysis (see Table 4 for detailed results) showed that Information, Clarity on Values and Support significantly explained variance in the mediating variable Decisional Conflict (a-path), in accordance with the Decisional Conflict model. However, the fourth variable, use of ROM feedback was not significantly associated with Decisional Conflict (a-path). Hierarchical multiple regression analysis conducted separately for the four independent variables (Block 1) with the mediator Decisional Conflict added in Block 2 (b-, c-and c’-path) revealed that Information, Clarity on values and Support could not explain a significant part of the variance in the primary outcome Symptom Change. In contrast, use of ROM feedback did explain a significant part of the variance in Symptom Change (*p* =.007), but it did not have a significant effect on the mediator Decisional Conflict in Block 1. Consequently no mediation analyses were applicable for the primary outcome (Fig. 4).

**Table 4:** Mediation analysis testing indirect effects of Information, Clarity about Values, Support and Use of ROM on Symptom Change, Symptom Severity and Patient Satisfaction through Decisional Conflict

	DV1: Symptom change			DV2: Symptom severity			DV3: Patient satisfaction		
Information									
	Coefficient	p-value	Bias -Corrected 95% CI	Coefficient	p-value	Bias -Corrected 95% CI	Coefficient	p-value	Bias -Corrected 95% CI
IV1 on DC (a)	0.693	<.001*	0.515 to 0.871	0.693	<.001*	0.515 to 0.871	0.693	<.001*	0.515 to 0.871
DC on outcome (b)	0.005	.604	−0.015 to 0.026	0.021	.026**	0.003 to 0.39	−0.034	.110	−0.075 to 0.008
IV1 on outcome (total effect c)	0.009	.162	−0.004 to 0.023	0.016	.013**	0.004 to 0.029	−0.032	.034	−0.062 to −0.002
IV1 on outcome (direct effect c')	0.006	.552	−0.014 to 0.025	0.002	.847	−0.016 to 0.019	−0.006	.775	−0.050 to 0.037
IV1 on outcome through: DC (ab)	0.003	.620	−0.160 to 0.167	0.014	.025**	−0.144 to 0.172	−0.024	.113	−0.263 to 0.215
Clarity about Values									
IV2 on DC (a)	0.726	<.001*	0.544 to 0.908	0.726	<.001*	0.544 to 0.908	0.726	<.001*	0.544 to 0.908
DC on outcome (b)	0.001	.932	−0.020 to 0.021	0.017	.070	−0.001 to 0.035	−0.044	.032	−0.084 to −0.004
IV2 on outcome (total effect c)	0.013	.071	−0.001 to 0.026	0.019	.004*	0.006 to 0.32	−0.024	.114	−0.054 to 0.006
IV2 on outcome (direct effect c')	0.012	.242	−0,008 to 0.032	0.007	.451	−0.011 to 0.025	0.008	.688	−0.033 to 0.050
IV2 on outcome through: DC (ab)	0.001	.920	−0.166 to 0.168	0.012	.066	−0.148 to 0.173	−0.032	.033	0.273 to 0.209
Support									
IV3 on DC (a)	0.720	<.001*	0.529 to 0.911	0.720	<.001*	0.529 to 0.911	0.720	<.001*	0.529 to 0.911
DC on outcome (b)	0.010	.328	−0.010 to 0.030	0.017	.057	−0.001 to 0.035	−0.022	.279	−0.063 to 0.019
IV3 on outcome (total effect c)	0.007	.345	−0.008 to 0.021	0.019	.005*	0.006 to −0.332	−0.038	.008*	−0.066 to −0.010
IV3 on outcome (direct effect c')	0.000	.975	−0.021 to 0.020	0.007	.461	−0.011 to 0.025	−0.022	.287	−0.062 to 0.019
IV3 on outcome through: DC (ab)	0.007	.320	−0.160 to 0.174	0.012	.070	−0.148 to 0.172	−0.016	.276	−0.252 to 0.221
Use of ROM feedback									
IV4 with confounder on DC (a)	−1.348	.559	−5.947 to −3.251	−1.348	.559	−5.947 to −3.251	−1.348	.559	−5.947 to −3.251
DC on outcome (b)	0.009	.194	−0.005 to 0.022	0.022	.001*	0.009 to 0.034	−0.038	.009*	−0.067 to −0.010
IV4 on outcome (total effect c)	−0.298	.007*	−0.512 to −0.083	−0.128	.248	−0.348 to −0.092	0.007	.975	−0.462 to −0.477
IV4 on outcome (direct effect c')	−0.290	.009*	−0.504 to −0.076	−0.110	.279	−0.311 to −0.092	−0.037	.868	−0.482 to −0.408
IV4 on outcome through + conf.: DC (ab)	−0.012	.593	−0.307 to 0.283	−0.030	0.560	−0.473 to 0.413	0.051	.566	−0.842 to 0.944

*Significant *p* <.0125 (Bonferroni correction), **marginally significant *p* <.025

#### Symptom Severity

We hypothesized that better application of SDM and the use of ROM feedback would also lead to lower Symptom Severity at T2, both directly and indirectly through a reduction in Decisional Conflict. As previously mentioned, the three independent variables - Information, Clarity about Values and Support - explained a significant part of the variance in the mediator Decisional Conflict. The hierarchical multiple regression analysis revealed that Feeling Informed, Having Clarity about one’s own values and Feeling Supported at T1, explained a significant part of the variance in the level of Symptom Severity at T2, also after applying the Bonferroni correction. Only the independent variable Information showed (in Block 2, b path) a marginally significant beta coefficient for the mediator (*p* =.026). Mediation occurs when the independent variable’s influence on the dependent variable is reduced after the mediator is controlled, this condition was met for Information. The beta coefficient declined from β = 0,016 (Block 1) to β = 0,002 (Block 2) and the significance level of this variable dropped from marginally significant to non-significant, indicating mediation. Results of the Sobel test confirmed a marginal significant indirect effect (ab path) of Decisional Conflict (*p* =.025), for the predictor Information on Symptom Severity (Fig. 4).

#### Patient Satisfaction

We hypothesized that better application of SDM and the use of ROM feedback would lead to increased Patient Satisfaction, both directly and indirectly through a reduction in Decisional Conflict. Hierarchical multiple regression analysis showed that feeling supported at T1 explained a significant part of the variance in Patient Satisfaction (*p* =.008) in Block 1. However, the coefficients for Feeling Informed, Clarity on Values and use of ROM feedback were not significant. After adding the potential mediator, Decisional Conflict, in Block 2, the effect of Support on Patient Satisfaction was no longer significant, while the b-path (the effect of DC on patient satisfaction) was not significant (Fig. 4).

#### Confounding and Interaction Effects

The mental health care organization was found as a confounder in the a-path between the independent variable use of ROM feedback and potential mediator Decisional Conflict (Adj. R^2^ =-.033, *F*(57) = 0.429, *β* = −1.348, *p* =.755). Controlling for this variable organization, the relationship between the use of ROM feedback and Decisional Conflict substantially changed (beta coefficients differ > 10%). Despite including the organization as a confounder, the effect of use of ROM feedback on Decisional Conflict remained non-significant (Fig. 4).

For the direct effects of predictors on outcomes, represented by the c ‘-path, we introduced an interaction term (diagnosis x predictor) in the second block of the regression analysis. The addition of this interaction term did not result in a significant increase in the explained variance.

## Discussion

This observational longitudinal study aimed to explore the (predictive) relationships between components of the shared decision making process (SDM) and the use of Routine Outcome Monitoring (ROM-feedback) with the primary outcome symptom change and secondary outcomes severity of symptoms and patient satisfaction during treatment. We explored the possible mediation effect of decisional conflict on these relationships. In this study we found no influence of the SDM process on symptom change. However, the results showed that the use of ROM feedback was associated with symptom change, with a greater reduction of symptoms when ROM feedback was used better. The SDM components information, clarity about values and support were associated with symptom severity later on in treatment: patients scoring more positive on these components showed less symptoms at a later moment in treatment. More experienced support during the SDM process was also associated with more patient satisfaction. Our hypothesis that the associations of the SDM process variables with outcome variables could be mediated by an indirect effect of decisional conflict was largely rejected. Only the effect of the independent variable information on symptom severity was mediated by decisional conflict. The primary diagnosis showed no impact on the relation between the SDM process and the use of ROM feedback with symptom change, symptom severity and patient satisfaction. The mental health care organization influences both the use of ROM feedback and the level of decisional conflict experienced by patients.

One of our findings is that patients who experienced a better SDM process, shown by feeling more informed, having greater clarity about values and feeling more supported, report less symptom severity at a later moment in treatment. For the variable feeling informed, this association was mediated by decisional conflict. It is understandable that feeling more informed leads to less decisional conflict (due to reduced uncertainty and improved decision quality) and, after accounting for the mediator decisional conflict, no longer has a direct association with symptom severity at a later point in treatment. However, the other SDM variables, clarity about values and feeling supported showed a direct association with lower symptom severity at later point in treatment, regardless of the decisional conflict score.

The influence of the SDM process aspects on symptom severity suggests that the way patients and clinicians communicate and work together (also called therapeutic relationship or working alliance) may be just as, if not more, important than the quality of the decision itself (decisional conflict). In the context of SDM, a strong trusting patient-clinician relationship forms a crucial foundation for the quality of this process, and a well-executed SDM can, in turn, strengthen this alliance (Eliacin et al., 2015b; Marchi et al., 2024; Priebe & Mccabe, 2008). The literature suggests that when patients and clinicians engage in shared communication and work together effectively, it leads to better treatment outcomes and higher satisfaction with mental health care (Eliacin et al., 2015b; Marchi et al., 2024; Priebe & Mccabe, 2008; Wampold, 2015). Our findings partly support this association, as we observed that more information, clarity about values, and support were associated with less symptom severity, and more support was linked to higher patient satisfaction. A possible explanation for the limited influence of decisional conflict might be that patient journeys in (specialist) mental health care usually involve a prolonged process of complex decision-making, and the mental condition of patients can hinder their decisiveness (Verwijmeren & Grootens, 2024). However, these uncertainties may become more manageable when patients trust their working relationship with the clinician (Eliacin et al., 2015a, b; Metz et al., 2023).

The theory behind SDM, as described by the Decisional Conflict Scale (Fig. 1), suggests that the way decisions are made influences how unsure patients feel about their options, which in turn affects the quality of their decision. According to the decisional conflict model, better SDM would be associated with less decisional conflict and, in turn, should lead to better treatment outcomes. However, our findings suggest that other associations may exist. In this study, the SDM process is directly associated with the outcome symptom severity, likely because these SDM process components contribute to a stronger therapeutic relationship. Patients also recognize SDM as a valued aspect of this therapeutic relationship (Eliacin et al., 2015a, b; Priebe & Mccabe, 2008). Moreover, a better therapeutic relationship is linked to better treatment outcomes, as described in a recent systematic literature review (Marchi et al., 2024). This review (Marchi et al., 2024) identifies the therapeutic relationship as one of the most significant predictors of treatment outcomes, emphasizing the collaborative process between patient and clinician in understanding the patient’s experiences, identifying their needs, and reaching consensus on treatment goals as a key component. Additionally, Wampold (2015) proposes a contextual model that highlights the role of ‘common factors’ such as patient expectations. This model describes the importance of the clinicians’ role in providing a fitting explanation for the patient’s condition. This helps the patient to believe they can take the necessary steps together to overcome or manage their issues. Central to this model is the dialogue between the patient and clinician about treatment goals, which is also part of the SDM process.

This literature on the therapeutic relationship and ‘common factors’ may explain why the experience of decisional conflict is not the most critical factor for better treatment outcomes and patient satisfaction. Process factors in SDM, such as information, clarity, support and the use of ROM feedback, can be seen as contributors to building a good therapeutic relationship or working alliance. These factors may play a significant role in positively affecting treatment outcome and patient satisfaction (Eliacin et al., 2015b; Jensen-Doss et al., 2020; Marchi et al., 2024). However, it is noteworthy that in this study, decisional conflict did mediate the relationship between feeling informed and reduced symptom severity. This might be understandable, since information about the content of a choice and its possible options is the first step in enabling a decision and being able to feel less uncertain and more comfortable about a decision which has to be made (Verwijmeren & Grootens, 2024).

The effect of using ROM feedback on symptom change aligns with previous research, which suggests that successful ROM implementation leads to more and earlier evaluations of, and communication about treatment goals resulting in better treatment outcomes, especially for patients who are not progressing as expected during treatment (Barkham et al., 2023; Carlier et al., 2012a, b; Jensen-Doss et al., 2020). Additionally, the finding that the organization was a confounder in the use of ROM feedback is consistent with other research indicating that ROM implementation is context-specific and depends on the organizations implementation process (Barkham et al., 2023; Metz et al., 2023).

### Strengths and limitations

One strength of this study is that it was conducted as a “real life” multi-center study, collecting data from a heterogenous and complex patient group with various diagnoses, representative of the population treated in Dutch ambulatory specialist mental health care settings. Although this study had an observational, non-randomized design, it was a longitudinal, prospective study carried out in clinical practice. It assessed the effects of implementing the SDM process and use of ROM feedback at the start of treatment in specialist outpatient mental health care settings over a three to six-month period. Conducting research in clinical practice is inherently challenging due to factors like incorrect referrals leading patients to move on to another organization or team, waiting lists between intake and start of treatment and patients who either cannot or choose not to continue participating due to their mental health status. Consequently, a relatively high number of drop-outs occurred after inclusion. In addition, because of the limited data and no randomized design, it was not possible to analyze and control for more than one confounder (mental health care organization) and one interaction variable (diagnosis). In general, the results need to be interpreted cautiously, because it was not a randomized design and we controlled for just two variables. Moreover, the limited sample size raises some uncertainty about the findings related to decisional conflict as a potential mediator. It remains unclear whether the limited mediation effect observed is due to a type 2 error caused by the small sample size or whether this study provides sufficient evidence of a genuine limited mediation effect of decisional conflict. Therefore, further research in a larger sample is recommended, ideally following patients until the end of their treatment. Monitoring patients in future research over a longer period is important for two main reasons: first, greater symptom changes observed at the end of treatment may reveal stronger associations and mediation effects; second, it allows for the investigation of the impact of the SDM application and the use of ROM feedback during treatment. Furthermore, patient satisfaction was measured by one general question, in follow-up research we recommend to clarify and specify this question for example into an evaluation of the collaboration with the clinician or the satisfaction about the result of treatment. Finally, it is important to note that the Cronbach’s alpha for one of the four independent variables, experienced support, was slightly below the acceptable threshold (α =.66), indicating insufficient reliability of this variable.

## Conclusion

This study suggests that the impact of the SDM process, consisting of feeling informed, having clarity about values, feeling supported and the use of ROM feedback, had more impact on the investigated outcomes, i.e. symptom change, symptom severity and patient satisfaction compared to the (un)certainty and quality of the decision itself, conceptualized by decisional conflict. Just the impact of the degree of feeling informed on symptom severity was mediated by decisional conflict. These findings support the notion that SDM involves much more than making a decision. It illustrates the importance of the SDM process with the patient-clinician working alliance as an important bedrock. Here, attention to understandable information, conversation about what a client considers important in life (values) and support from loved ones and clinician(s) is essential in a good SDM-process. Additionally, a good SDM process promotes the working alliance.

However, to provide more evidence for these initial findings, further research should focus on the role and impact of decisional conflict in a larger sample over a longer period of time.
